# Improving measures of women’s and girls’ sexual empowerment

**DOI:** 10.2471/BLT.24.291975

**Published:** 2024-10-02

**Authors:** Anna E Kågesten, Miranda van Reeuwijk, Caroline Moreau

**Affiliations:** a Department of Global Public Health, Karolinska Institutet, Tomtebodavägen 18A, 17177 Stockholm, Sweden.; b Rutgers, Utrecht, Kingdom of the Netherlands.; c Department of Population, Family and Reproductive Health, Johns Hopkins Bloomberg School of Public Health, Baltimore, United States of America.

Two decades ago, the World Health Organization (WHO) formulated its working definition of sexual health as more than just the absence of disease, underscoring the need for a positive approach to sexuality that recognizes its link with human rights and power dynamics.^1^ This definition includes the notion that sexual health is fundamentally tied to empowerment, whereby individuals can make informed decisions related to their sexuality, including having pleasurable and safe sexual experiences. Yet, such an affirmative approach is far from true for many women and girls globally, as their sexuality remains a sociocultural and political battleground.^2^^,^^3^

At the midpoint of the *2030 Agenda for sustainable development*, the world is falling short of reaching the sustainable development goal (SDG) of empowering women and girls, including ensuring their universal access to sexual and reproductive health and rights.^4^ Available data indicate that only half of married women aged 15–49 years can decide about their sexual relations and reproductive health, with large disparities ranging from an estimated 47% of women in sub-Saharan Africa to an estimated 82% in Europe and North America.^4^

However, these data only capture a fraction of women’s sexual empowerment, as common indicators from surveys such as the Demographic and Health Surveys (DHS) lack depth and specificity.^5^^,^^6^ Recent efforts have led to the development of more conceptually grounded indicators of empowerment,^6^^,^^7^ but most emphasize reproductive rather than sexual aspects. Additionally, few organizations have formulated definitions of sexual empowerment that guide theories of change and programme evaluations, although such empowerment is central to many sexual and reproductive health rights programmes.^3^ This gap reflects the sociopolitical sensitivity of promoting women’s sexual empowerment and the lack of consensus on how to define and measure it.

In this article, we argue that improving measures of women's and girls’ sexual empowerment is not just an academic exercise but a practical need to track progress towards advancing gender equality and sexual and reproductive health and rights as part of global development efforts.

## Defining women’s sexual empowerment

Most scholars agree that women’s empowerment, including sexual empowerment, is a dynamic and multidimensional construct.^2^^,^^3^^,^^6^^,^^8^^,^^9^ While its specific conceptualization varies, consensus exists that empowerment is a process shaped by an individual’s agency or ability to voice, choose and act on one’s goals, through the mobilization of resources (both tangible and intangible) and interactions with institutions to reach those goals.^2^^,^^3^^,^^5^^,^^6^^,^^8^^,^^10^^,^^11^

This definition implies that sexual empowerment involves, first, the availability of options and alternatives for women and girls regarding if, when and with whom to be sexually active, free from social pressures and structural constraints (existence of choice). Second, the ability to make decisions from these options, supported by confidence, knowledge, negotiation skills and external support (exercise of choice). Third, the realization of one’s goals and preferences (achievement of choice).^2^^,^^6^^,^^10^ This process is intrinsically linked with access to resources and structures such as education, employment and social norms that shape women’s status and power.^2^^,^^3^^,^^10^^,^^11^ Thus, beyond the individual, sexual empowerment involves collective action to navigate broader social and power structures enabling women to voice and act on their sexual choices.^2^^,^^6^^,^^11^

A commonly discussed issue is whether empowerment is best measured subjectively or objectively, that is, who decides who is empowered? Some scholars define sexual empowerment as related to internal subjective perceptions of agency and sense of control – often referred to as power-to. In contrast, others argue that such an internal sense of empowerment fails to achieve its objective if an individual has limited power and autonomy relative to their partners or broader social constraints – sometimes referred to as power-over.^11^ Thus, while measures that capture women’s perceived autonomy to choose is important from a human rights-based perspective, such measures may imply that individuals are always in control (for example by avoiding unwanted sex), which may not be the case.^2^^,^^3^ The conceptual understanding of empowerment as a process bridges this divide, moving from the existence of choice emphasizing the power to define one’s goals, to exercising power-over to reach these goals.^2^^,^^3^^,^^11^

## Current measures and shortcomings

Women's and girls’ sexual empowerment has been explored through a variety of indicators. Some indicators focus on specific components, such as sexual decision-making and autonomy – for example, the proportion of women who make informed decisions over their sexual relations. Others examine outcomes that reflect a lack of choice, such as incidence of sexual violence.^4^ Additionally, some indicators address decisions related to sexual activity, such as contraceptive use and reproductive health care, as part of broader measures of sexual and reproductive health empowerment.^6^^–^^8^^,^^12^ Other measures consider elements that contribute to sexual empowerment, such as economic empowerment, but do not explicitly address sexuality or sexual and reproductive health and rights.^10^

Below we summarize some of the shortcomings of existing tools, along with some notable exceptions.

First, most existing measures were developed among married adult women in a single country, either in high-income contexts or in sub-Saharan Africa and South-East Asia.^7^^,^^12^ As such, they often lack cross-cultural validation for use in other settings, failing to capture cultural and social norms that shape women’s sexual autonomy and decision-making, especially in low- and middle-income countries.^8^^,^^9^ Promising examples that have attempted to address this concern include the sexual and reproductive empowerment scale, initially developed for adolescent girls and young women in the United States of America,^8^ which was later adapted and validated in Kenya using qualitative methods.^7^ In addition, the Women’s and girls’ sexual and reproductive health empowerment index was developed in four diverse settings in sub-Saharan Africa using mixed-methods to capture common expressions of empowerment across cultures.^6^^,^^9^

Second, existing tools tend to use proxy measures that only indirectly touch on sexuality. For example, a four-item sexual empowerment scale in Bangladesh^12^ relied on DHS questions around condom negotiation, sexual violence and wife-beating norms as proxies, with the authors highlighting the need to include measures of women’s ability to express their sexuality and sexual needs.

Third, existing measures are mostly unidimensional, failing to distinguish between the existence and exercise of sexual choices. A girl might feel empowered to have sexual feelings and desires, but lacks the ability to act on these choices given societal expectations or partner pressures. The Women’s and girls’ sexual and reproductive health empowerment measure, which integrates this distinction, demonstrates the added value of considering both dimensions in predicting volitional sexual activity.^6^^,^^9^ Beyond issues of dimensionality, most measures centre on individual-level attributes of sexual empowerment, while few tap into collective empowerment to mobilize resources and create broader opportunity structures to help women achieve their sexual goals.

Fourth, most measures do not consider sexual empowerment as a developmental process that intensifies during adolescence and continues throughout the life course.^2^^,^^11^ This gap highlights the need for longitudinal approaches to assess sexual empowerment in adolescence and over time.

## Moving forward

Drawing from these insights and current best practices, we highlight five recommendations for future sexual empowerment measurement development. 

First, the measurement should be conceptually grounded. Comprehensive measures of sexual empowerment should be guided by theoretical frameworks that reflect individual and collective agency, access to resources, and structural and institutional aspects.^6^^,^^9^^,^^10^ Such frameworks can also inform the theories of change for sexual and reproductive health and rights programmes.^2^ In addition, realist evaluation approaches can be useful to understand contextual influences, and how and under which circumstances interventions can trigger changes in empowerment.

Second, contextual relevance is key. While frameworks provide a structure, measures should be developed with a deep understanding of the specific contexts where they are to be applied. Participatory research methods, where women and girls are actively involved in measurement development and validation, can help ensure that tools are culturally meaningful and practically applicable.

Third, the development of sexual empowerment measurement should use longitudinal, mixed-methods approaches. Combining longitudinal quantitative surveys with qualitative methods can provide rich insights into women's and girls’ preferences and ability to navigate within the constraints of their social environment, and how they lose or gain sexual empowerment across the life course.

Fourth, the measurement should ensure diversity and adopt intersectional approaches. Sexual empowerment measures should be inclusive of all women and girls, including younger and unmarried women who face greater societal stigma in many low- and middle-income countries,^2^^,^^7^ and for whom questions related to partner dynamics are less applicable. Additionally, opportunities to make informed choices are not equally available to all, calling for intersectional perspectives recognizing how factors such as age, race, socioeconomic status, education and disability intersect to shape how women and girls exercise agency and access resources.

Fifth, the measurement should use dynamic tools that leverage technology. Digital platforms and mobile technologies can serve to support these measurement innovations, involving repeated measures, capable of evolving with changing circumstances. The performance monitoring and action data used to develop the Women’s and girls’ sexual and reproductive health empowerment index^6^ shows promise in advancing measurement innovations, given its multinational longitudinal design to measure both existence and exercise of choice as different components of empowerment.

## Conclusion

Sexual empowerment must be measured to drive meaningful and lasting change. The post-2030 agenda period presents an opportunity to do so by rethinking and optimizing the way we measure and promote sexual empowerment as a critical, but often overlooked, component of women's and girls’ empowerment. These tools should be comprehensive, context-specific, and capable of capturing both individual and collective dimensions of empowerment as well as the influence of broader social and structural factors. Such an understanding is crucial for informing the design and evaluation of effective sexual and reproductive health and rights interventions and policies that empower women and girls and contribute to broader societal progress.
